# Supplementary material for: eQTL in diseased colon tissue identifies novel target genes associated with IBD
Source: bioRxiv. 2024 Oct 17:2024.10.14.618229. Preprint. [Version 1] doi: 10.1101/2024.10.14.618229 (PMC11507739; doi:10.1101/2024.10.14.618229)
Supplement: 1 [file NIHPP2024.10.14.618229V1-supplement-1.pdf]

bioRxiv preprint doi: https://doi.org/10.1101/2024.10.14.618229; this version posted October 17, 2024. The copyright holder for this preprint (which was not certified by peer review) is the author/funder, who has granted bioRxiv a license to display the preprint in perpetuity. It is made available under aCC-BY 4.0 International license.

Supplementary Figures

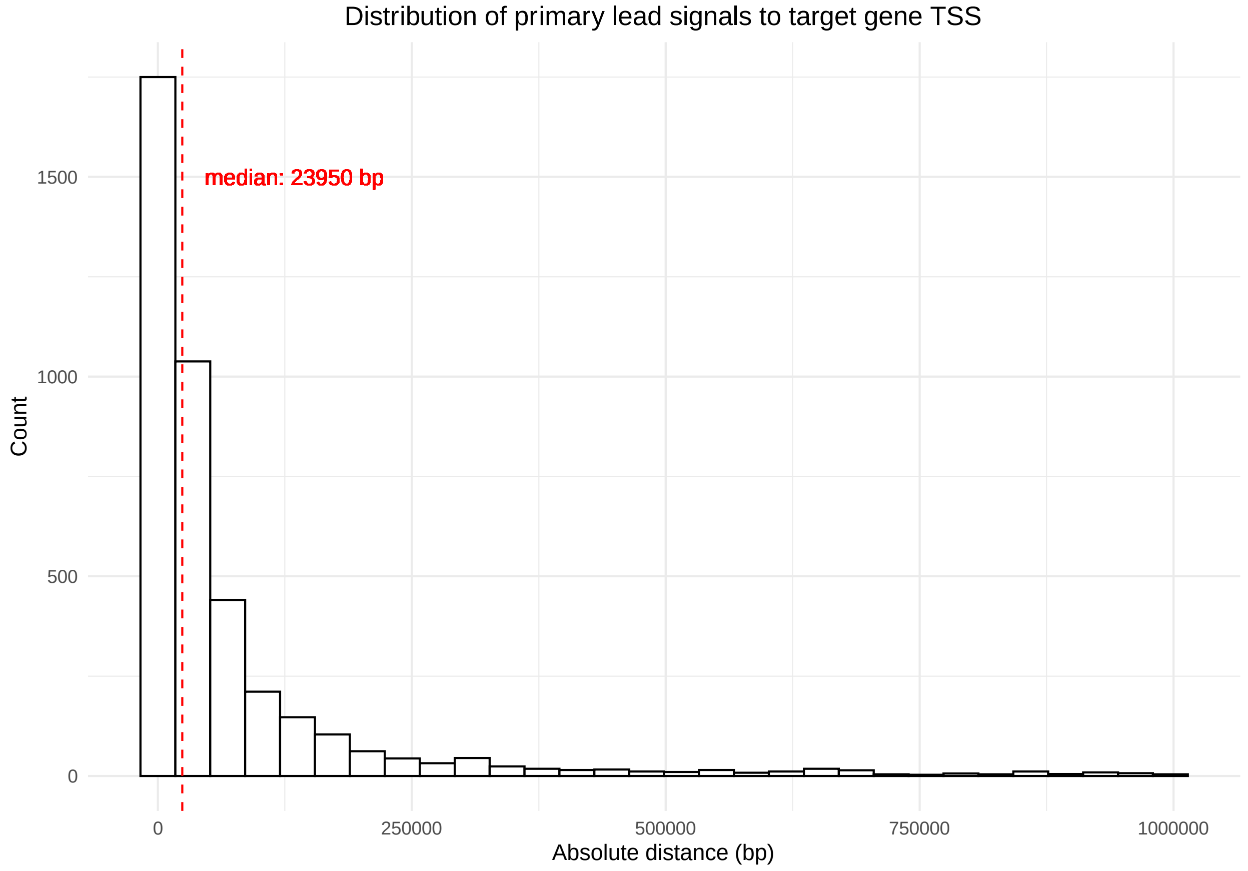

Supplementary Figure 1 Primary lead eQTL signals are proximal to target gene transcription start site (TSS).

bioRxiv preprint doi: https://doi.org/10.1101/2024.10.14.618229; this version posted October 17, 2024. The copyright holder for this preprint (which was not certified by peer review) is the author/funder, who has granted bioRxiv a license to display the preprint in perpetuity. It is made available under aCC-BY 4.0 International license.

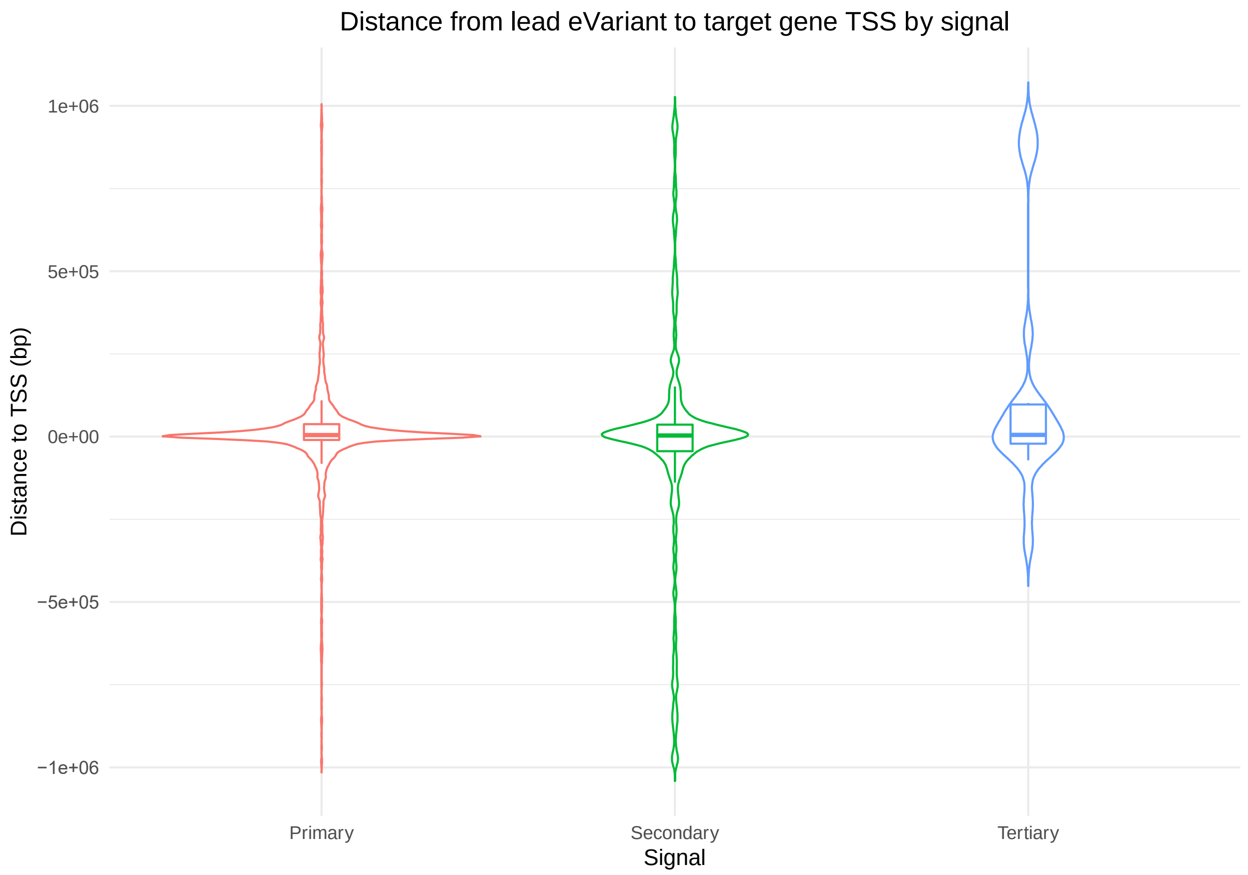

Supplementary Figure 2 Distribution of distance from lead eVariant to target gene TSS by signal rank.

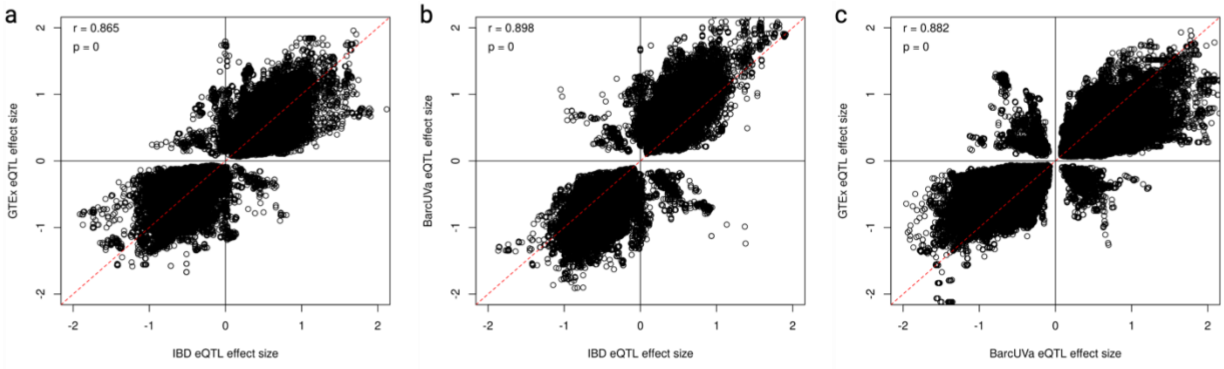

Supplementary Figure 3 Effect Size Correlation between all significant shared eVariant-eGene pairs.

bioRxiv preprint doi: https://doi.org/10.1101/2024.10.14.618229; this version posted October 17, 2024. The copyright holder for this preprint (which was not certified by peer review) is the author/funder, who has granted bioRxiv a license to display the preprint in perpetuity. It is made available under aCC-BY 4.0 International license.

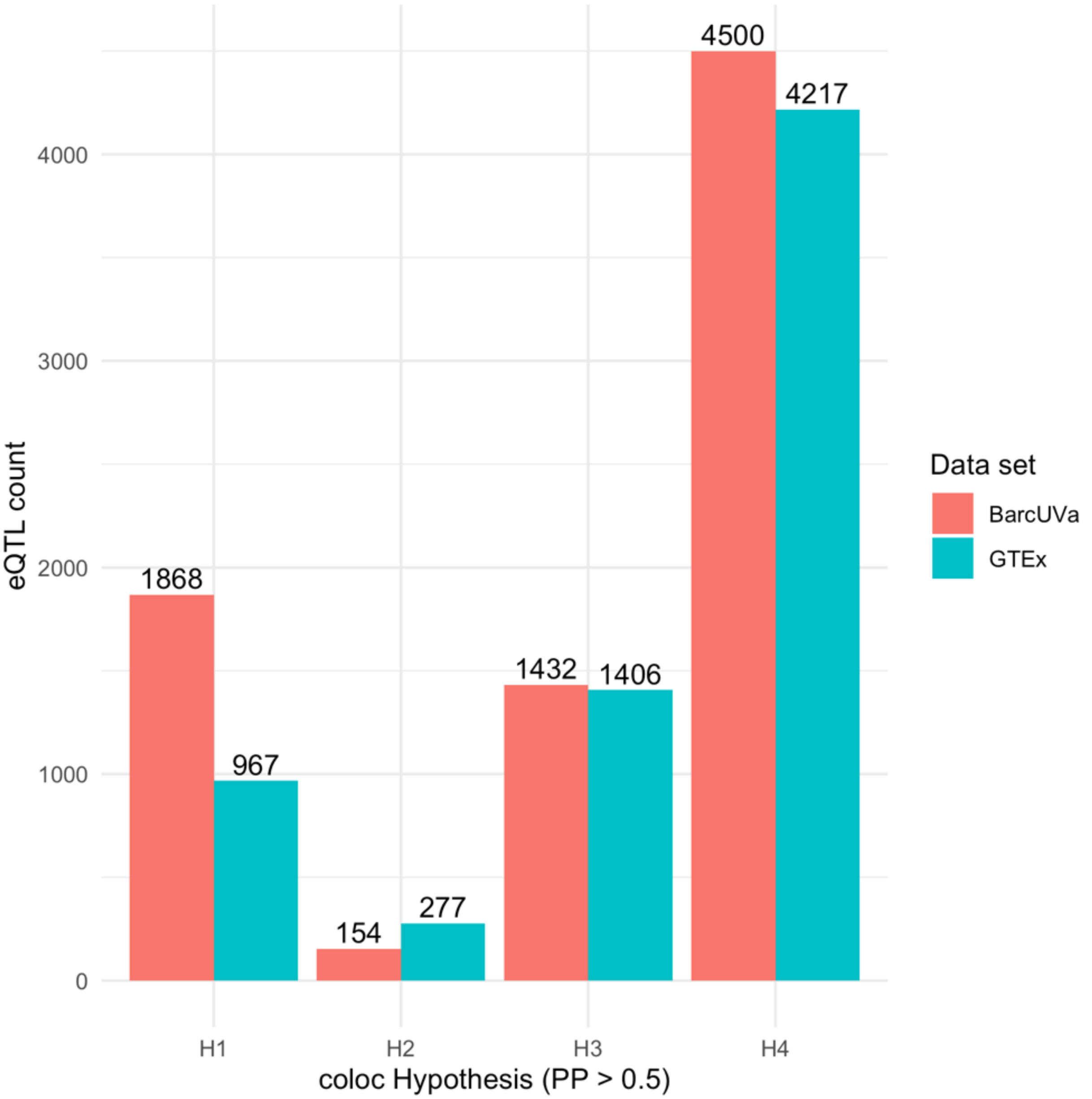

Supplementary Figure 4 Bar plot of GTEx eQTL-BarcUVa eQTL colocalization results by coloc hypothesis (PP > 0.5): H1, significant signal detected in reference data set only (self); H2, significant signal detected in comparison data set only; H3, significant signal detected in both data sets but different causal variants; H4, significant signal detected in both data sets, same causal variant.

bioRxiv preprint doi: https://doi.org/10.1101/2024.10.14.618229; this version posted October 17, 2024. The copyright holder for this preprint (which was not certified by peer review) is the author/funder, who has granted bioRxiv a license to display the preprint in perpetuity. It is made available under aCC-BY 4.0 International license.

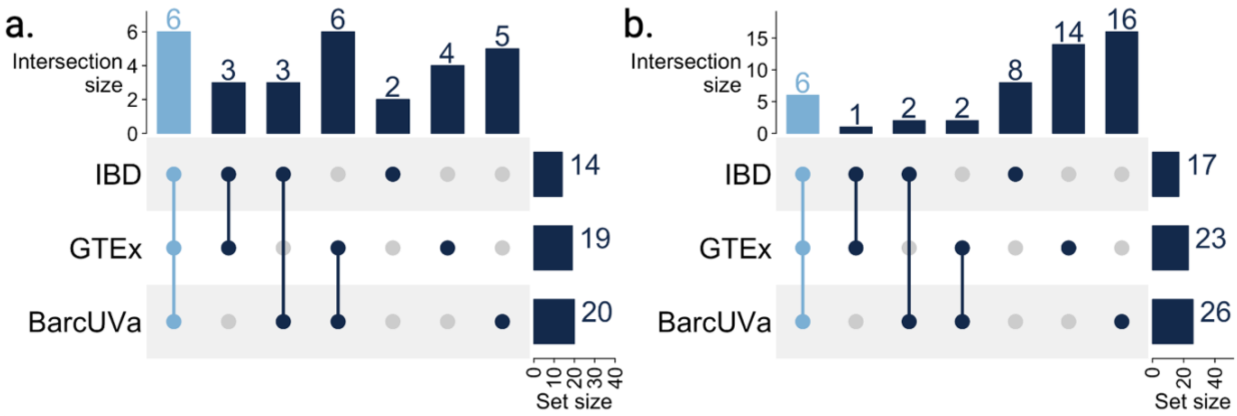

Supplementary Figure 5 Sharing of colocalization results for newly reported IBD GWAS loci. a. UpSet plot showing overlap of newly reported colocalizing GWAS loci across studies. b. UpSet plot showing overlap of eGenes that colocalize with newly reported IBD GWAS loci across studies.

bioRxiv preprint doi: https://doi.org/10.1101/2024.10.14.618229; this version posted October 17, 2024. The copyright holder for this preprint (which was not certified by peer review) is the author/funder, who has granted bioRxiv a license to display the preprint in perpetuity. It is made available under aCC-BY 4.0 International license.

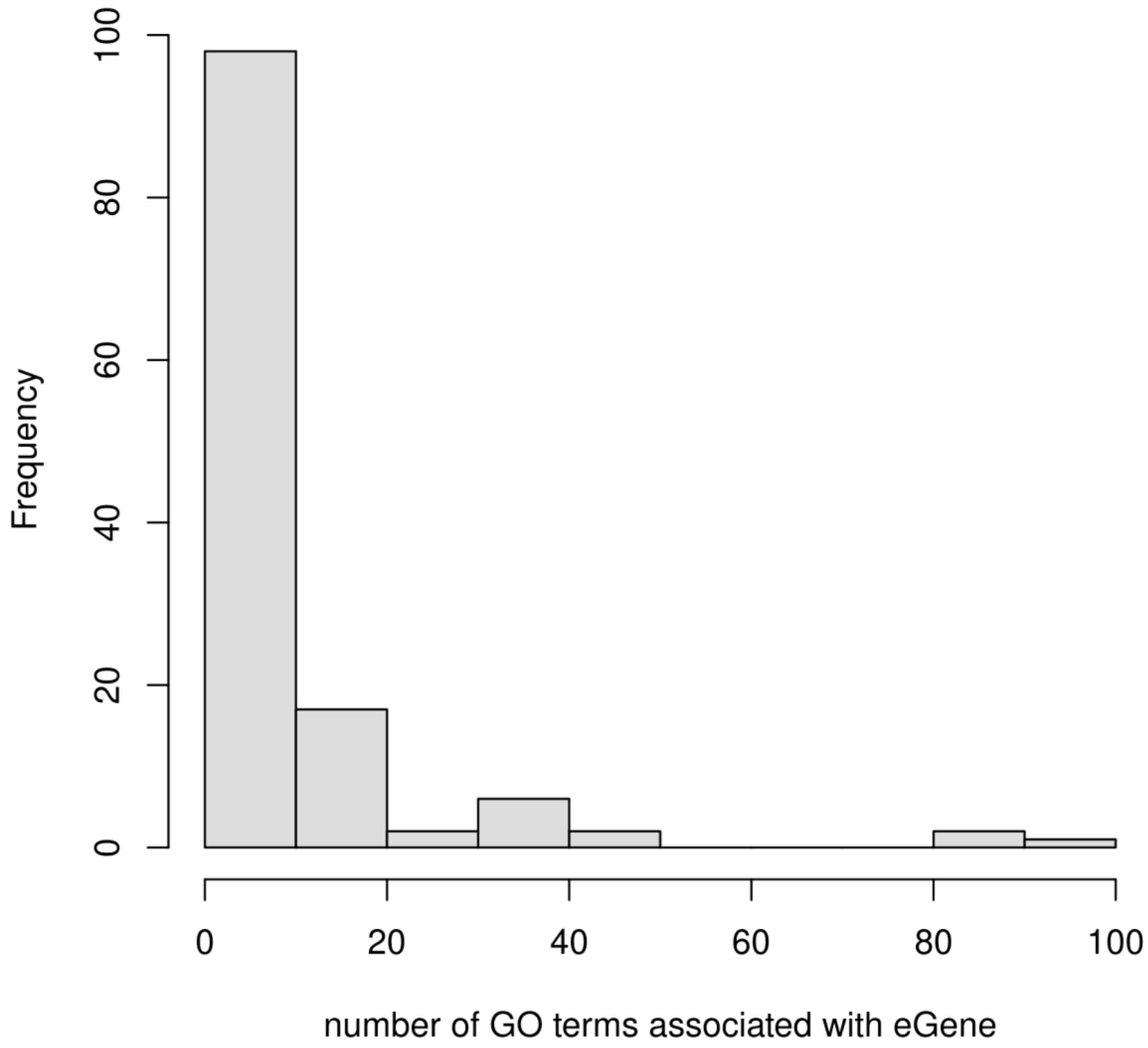

Supplementary Figure 6 Histogram of the number of GO terms associated with colocalizing eGenes.

bioRxiv preprint doi: https://doi.org/10.1101/2024.10.14.618229; this version posted October 17, 2024. The copyright holder for this preprint (which was not certified by peer review) is the author/funder, who has granted bioRxiv a license to display the preprint in perpetuity. It is made available under aCC-BY 4.0 International license.

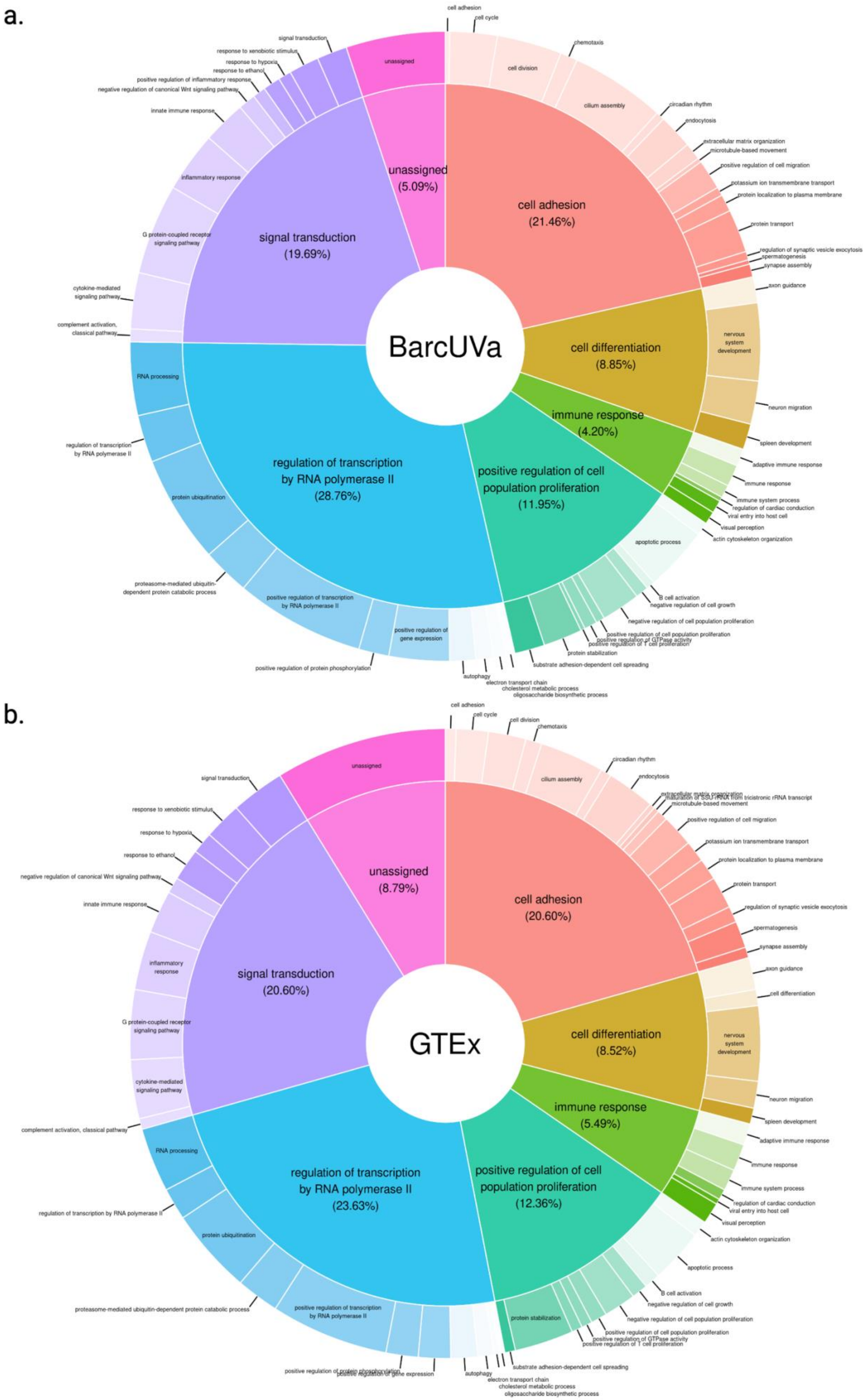

bioRxiv preprint doi: https://doi.org/10.1101/2024.10.14.618229; this version posted October 17, 2024. The copyright holder for this preprint (which was not certified by peer review) is the author/funder, who has granted bioRxiv a license to display the preprint in perpetuity. It is made available under aCC-BY 4.0 International license.

C

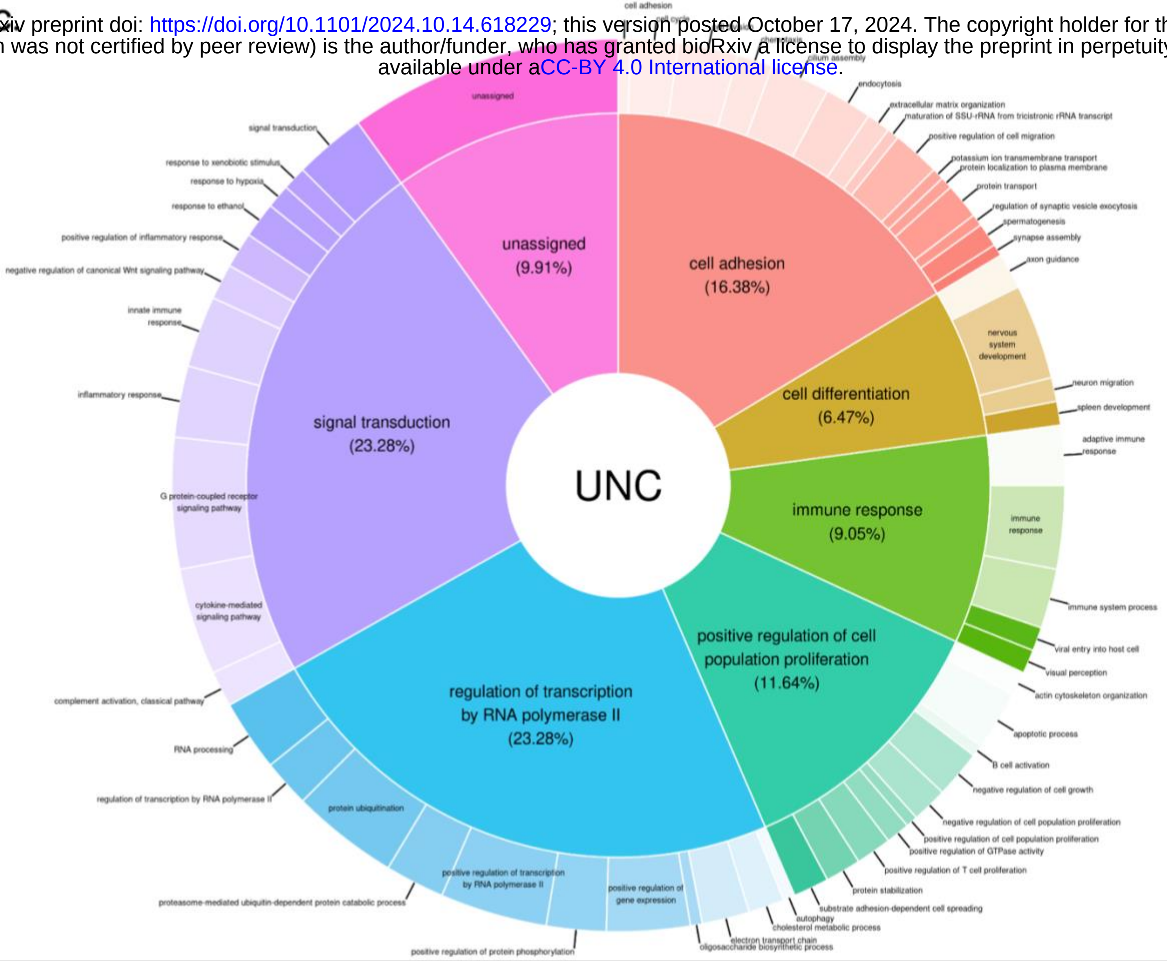

Supplementary Figure 7 Pie-Donut charts representing clustered GO terms associated with colocalizing eGenes. Inner pie slices show the proportion of colocalizing eGenes that map to primary clusters. Outer slices represent proportion of colocalizing eGenes that map to secondary sub-clusters.

bioRxiv preprint doi: https://doi.org/10.1101/2024.10.14.618229; this version posted October 17, 2024. The copyright holder for this preprint (which was not certified by peer review) is the author/funder, who has granted bioRxiv a license to display the preprint in perpetuity. It is made available under aCC-BY 4.0 International license.

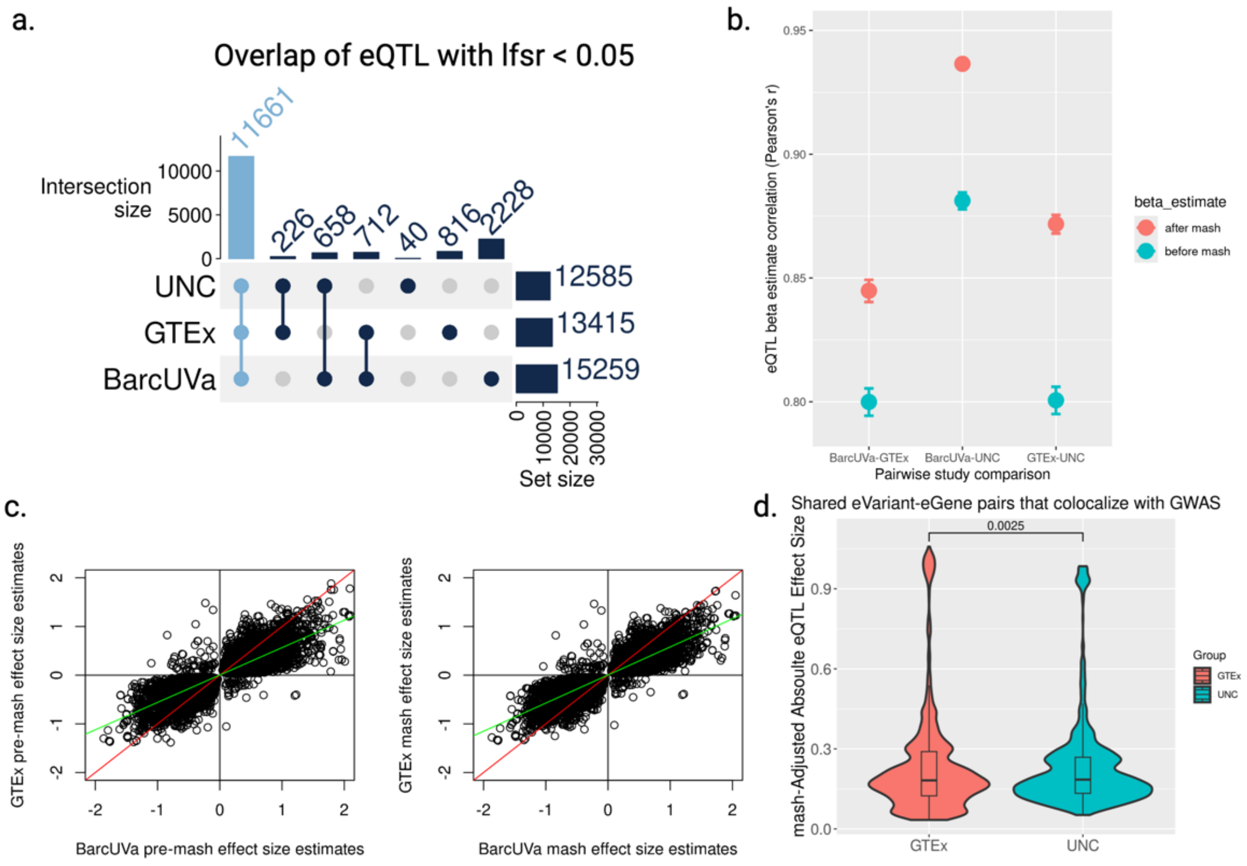

Supplementary Figure 8 Mash results. a. UpSet plot of overlap for eQTL with lfsr < 0.05. The 11,661 overlapping eQTL highlighted in Carolina blue were used to generate data in panels b and c. b. Scatterplot of correlation estimates for pairwise eQTL study comparisons of effect size estimates before and after applying mash. Vertical bars represent the 95% confidence interval for each estimate. c. Effect size correlation for non-IBD eQTL before and after applying mash. The red line represents the (0,1) intercept. The green line represents the regression fit. d. Violin plots of absolute eQTL effect size distributions for the 6315 shared eVariant-eGene pairs that colocalize with GWAS loci. p-value calculated from the Mann-Whitney U statistic.

bioRxiv preprint doi: https://doi.org/10.1101/2024.10.14.618229; this version posted October 17, 2024. The copyright holder for this preprint (which was not certified by peer review) is the author/funder, who has granted bioRxiv a license to display the preprint in perpetuity. It is made available under aCC-BY 4.0 International license.

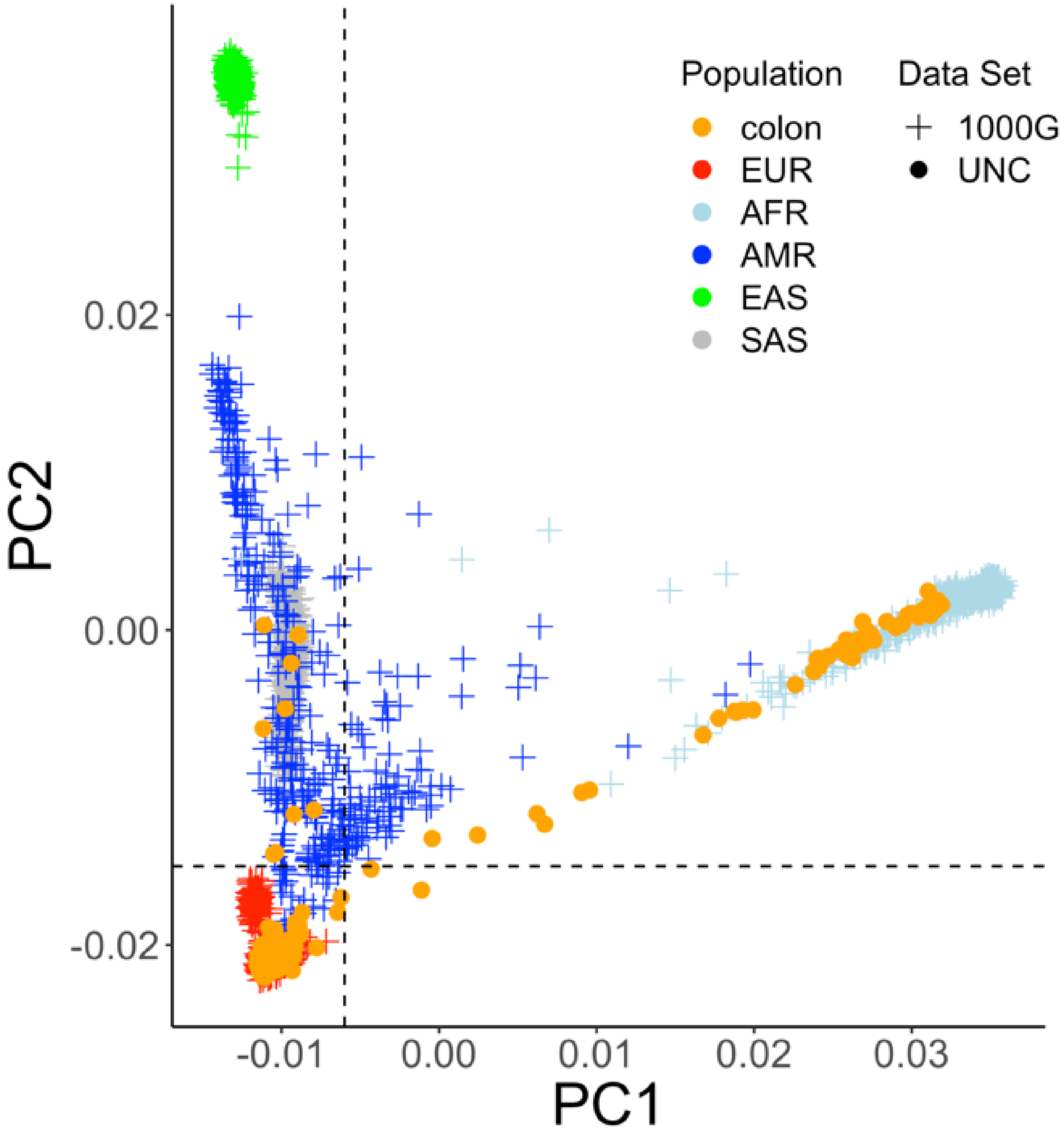

Supplementary Figure 9 IBD patients used in this study are of primarily inferred European genetic ancestry.

bioRxiv preprint doi: https://doi.org/10.1101/2024.10.14.618229; this version posted October 17, 2024. The copyright holder for this preprint (which was not certified by peer review) is the author/funder, who has granted bioRxiv a license to display the preprint in perpetuity. It is made available under aCC-BY 4.0 International license.

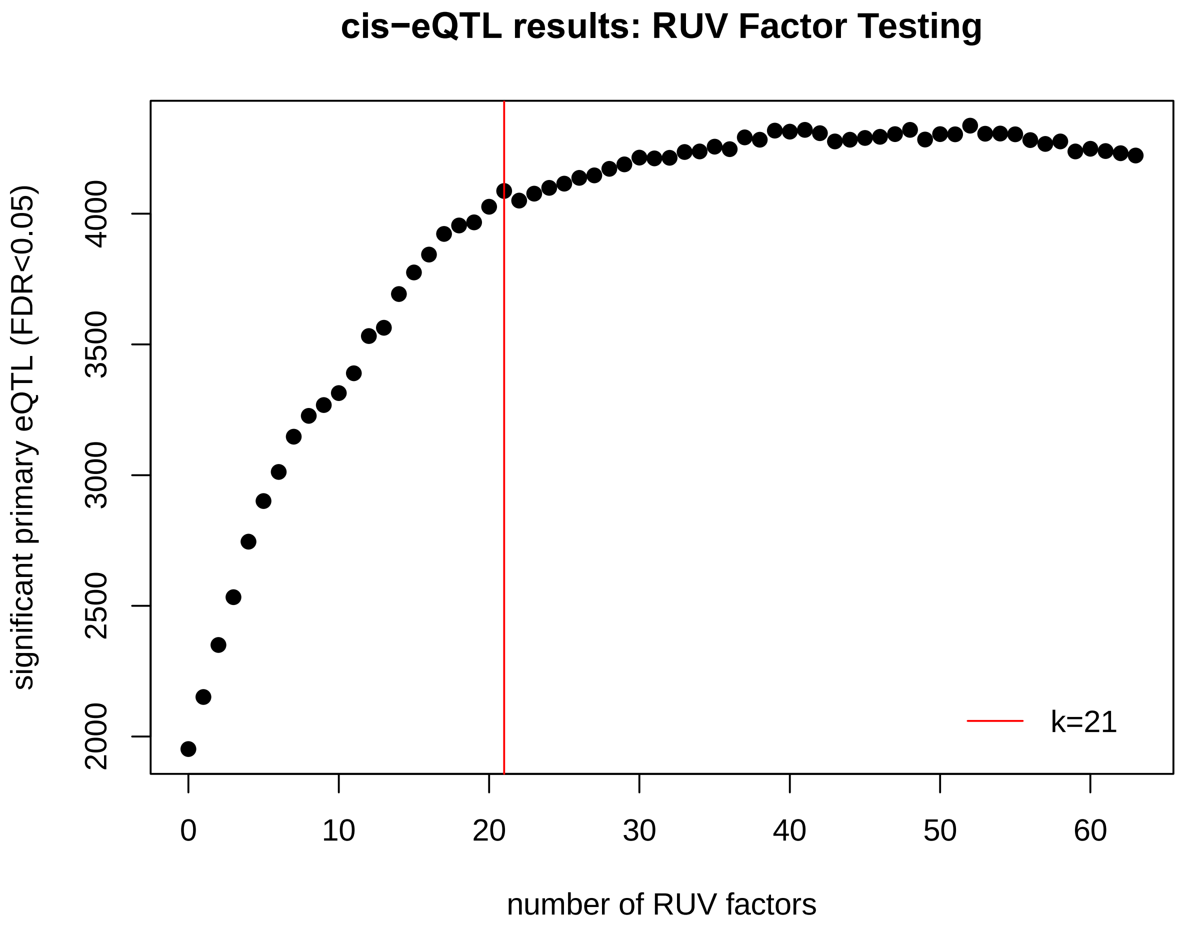

Supplementary Figure 10 Scatter plot of the number of significant primary lead eQTL mapped using IBD tissue using increasing number of RUV factors included in eQTL model. The red vertical line indicates the point of maximum curvature detected by kneedle and the final number of RUV factors included in the eQTL model.
